# Crystal-momentum dispersion of ultrafast spin change in fcc Co

**DOI:** 10.1038/srep05010

**Published:** 2014-05-23

**Authors:** M. S. Si, J. Y. Li, D. Z. Yang, D. S. Xue, G. P. Zhang

**Affiliations:** 1 Key Laboratory for Magnetism and Magnetic Materials of the Ministry of Education, Lanzhou University, Lanzhou 730000, China; 2 Department of Physics, Indiana State University, Terre Haute, Indiana 47809, USA

## Abstract

Nearly twenty years ago, Beaurepaire and coworkers showed that when an ultrafast laser impinges on a ferromagnet, its spin moment undergoes a dramatic change, but how it works remains a mystery. While the current experiment is still unable to resolve the minute details of the spin change, crystal momentum-resolved techniques have long been used to analyze the charge dynamics in superconductors and strongly correlated materials. Here we extend it to probe spin moment change in the entire three-dimensional Brillouin zone for fcc Co. Our results indeed show a strong spin activity along the Δ line, supporting a prior experimental finding. The spin active pockets coalesce into a series of *spin surfaces* that follow the Fermi surfaces. We predict two largest spin change pockets which have been elusive to experiments: one pocket is slightly below the Δ line and the other is along the Λ line and close to the L point. Our theory presents an opportunity for the time-, spin- and momentum-resolve photoemission technique.

Crystal momentum-resolved techniques have been instrumental to understanding electronic and magnetic properties in a variety of materials from high Tc superconductors^1^ to magnetic materials. Angle-resolved photoemission (PE) is an example, where one can examine charge and lattice dynamics^2^ or pure electron dynamics in antiferromagnets^3^ along a few selected crystal momentum lines. This technique has now been extended to x-ray energy regions where its crystal-momentum resolving power is augmented by the element specificity. The first time-resolved photoemission measurement of the ultrafast spin dynamics in Co was demonstrated over a decade ago^4^, but it was not momentum-resolved. The situation did not improve much up to now^5^, with a few exceptions^6^. Majority of laser pulse durations are too long to fully resolve the spin polarization change at the earliest stage. Without any theoretical guidance, it is difficult to prove that the spin polarization really reflects the spin moment change in a sample. This is because experiments measure the spin polarization of those ejected electrons, not those electrons in the sample, while one is interested in the spin moment change of those electrons left behind. As a result, the response of spin-polarized photoelectrons to the laser pulse may differ from those electrons which contribute demagnetization in femtomagnetism. Weber *et al.*^7^ recently even showed different demagnetization times for the time- and spin-resolved photoemission and the time-resolved magneto-optics. This explains why Pickel *et al.*^6^ could only examine the momentum-resolved spin-orbit hybridization, not momentum-resolved spin moment changes which are a focus in femtomagnetism^8^,^9^,^10^.

Despite of those uncertainties in PE, crystal momentum-resolved techniques can provide a microscopic picture momentum by momentum and allow a direct comparison with theoretical calculations. Some of the latest experiments reported in a recent conference may represent a major breakthrough. Ross *et al.*^11^ showed that it is possible to enhance the experimental signal by an order of magnitude by collecting cascade electrons. In sharp contrast to almost all the prior studies, here the spin moment changes are crystal momentum-resolved, in a similar fashion as done for the charge dynamics. Undoubtedly, this will open a new frontier in femtomagnetism. A theoretical investigation is imperative.

Face-centered cubic cobalt is a conventional 3 *d* ferromagnetic transition metal, and its magnetic properties have been thoroughly studied over two decades^12^. In the realistic calculation, we use the experimental lattice constant of 3.549 Å^13^. The obtained magnetic spin moment of Co is about 1.66 *μ*_B_, which agrees well with the previous theoretical value 1.64 *μ*_B_^14^. While more advanced theoretical methods are available for smaller systems^15^, at present it is difficult, if not impossible, to use them to compute dynamical processes for a solid. Our theoretical calculation is based on the density functional theory (DFT) using the full potential augmented planewave method as implemented in the WIEN2k code^16^,^17^. For the exchange-correlation functional we use the generalized gradient approximation (GGA) in the form of Perdew-Burke-Ernzerhof (PBE) functional^18^. Our spin moment and band structure fully agree with the previous experimental results and theoretical results^12^,^19^,^20^,^21^. In the laser excitation both the Kohn-Sham (KS) ground and excited states are important; to accurately take into account the delocalized nature of excited states, a Muffin-tin radius *R_mt_* is set to 2.36 a.u. and a large product of the Muffin-tin radius and the planewave cutoff, i.e., *R_mt_K_max_*, is set to 9.5. Thus, more basis functions are included to accurately describe high lying states at the laser photon energy. The number of the basis functions is 95. After solving the KS equation self-consistently, we compute the optical transition^22^,^23^ and spin matrices^17^, and get ready for the laser-induced spin dynamics.

Although significant work has been devoted to the understanding of ultrafast demagnetization, for example electron-phonon scattering theory^24^,^25^, superdiffusive spin transport mechanism^26^,^27^, and others^28^,^29^,^30^, the microscopic origin is still unclear. Alternatively, we propose a demagnetization mechanism based on the electronic transitions, where the spin-orbit coupling (SOC) plays a role in the spin moment change. Under the influence of a laser pulse, electrons are excited out of the Fermi sea. The entire electron dynamics can be quantitatively computed through the Liouville equation for the density matrix *ρ* at each **k**^31^, 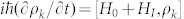. This is a coupled equation involving transitions between all the band states and is solved numerically. Here *H*_0_ is the system Hamiltonian, and *H_I_* is the interaction between the laser and the system, i.e., 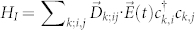, where 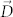 is the dipole operator, and 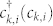 is the electron creation (annihilation) operator for band *i* at **k**. The laser field is 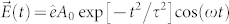, where *ê* is the laser polarization direction, *A*_0_ is the field amplitude, *ω* is the laser frequency, *t* is the time and *τ* is the laser pulse duration. Once we obtain the density matrix, the time-dependent spin moment can be computed by tracing over the product of the density matrix and spin matrix. The major strength of our simulation is that we realistically include the interaction between the laser system and the ferromagnetic sample. There is no fitting parameter in our model. The major weakness is that the electron-electron interaction is not included. There are two significant findings of our study. First, although there are many excitations among electrons, only those electrons, whose initial and final spin moments are different, can contribute strongly to the total spin moment change. This process is mediated by SOC. Second, we demonstrate that there are large pockets of the isosurfaces of spin moment change in the Brillouin zone, which are accessible to the time- and crystal momentum-resolved photoemission experiments.

We emphasize that the dipole matrix elements are not updated during our time dependent simulations. This is different from the standard TD-DFT where the dipole element is updated dynamically. Unfortunately, a TD-DFT calculation is extremely difficult if it is not impossible because nearly half million *k* points are needed to converge the spin moment change^17^. This situation is unlikely to change in the near future. Our method relies on the ground state property of DFT, and is a compromise between the accuracy and the efficiency. Naturally, we are fully aware of the limitation of our formalism. First, its validity has been checked against a correlated model^32^. Second, we also checked against the quantum chemistry calculation in a small system^15^, which is carried out by the Gaussian package. The agreement is very good. Third, we carried out a calculation using the TD-DFT in a small system and then compared our results. We find that the major difference in the electric polarization appears at the earliest time, where the exchange kernel undergoes the major change. The difference is that in our method there is a smaller delay between the laser peak and the system's response, but the TD-DFT has a slightly larger delay (larger than 1–5 fs). All the subsequent evolution is nearly identical. Since the spin change occurs on a few hundred fs, this short delay does not affect our results significantly. When a laser pulse becomes too strong, our formalism becomes less accurate, since the laser perturbs the system strongly.

It is well known that photon energy plays a vital role in femtomagnetism. This is because it acts as a selector of electron transition window, which subsequently dominates the spin moment change associated with bands. Thus, we optimize the photon energy where the pulse duration of 12 fs and the field amplitude of 0.05 V/Å are fixed during the optimization process. Our results show that the spin moment change depends critically on the photon energy. When the photon energy is about 1.9 eV, the laser pulse generates the maximum spin moment reduction. The obtained demagnetization curve is similar to that obtained in fcc Ni^33^ (not shown for brevity). The spin moment first drops sharply and then reaches its minimum at 23.6 fs. After that the spin recovers itself and oscillates around an average value of 1.638 *μ*_B_. In comparison with the result in fcc Ni, we notice that the first minimum appears at a later time which originates from the weaker SOC in fcc Co.

In order to reveal further insights into the spin moment change, we disperse the time-averaged spin change (after 90 fs) in the first Brillouin zone. While there are a few isolated hot spin spots^34^, mojority of the spots with large spin moment change coalesce into larger segments. Their intensity map is defined as the spin surface, in analogy to the Fermi surface. There are three reasons why these spin surfaces exist. First, due to the laser photon energy, only a limited number of unoccupied conduction bands are accessible from the occupied states, the top of which are at the Fermi level. In our case, these bands appear about 2 eV (our photon energy) above the occupied band states. Those bands form a surface. Second, among those unoccupied bands, a small portion is excited strongly because their transition energy matches the photon energy. Third, among those excited bands, only those bands that have a large spin moment change can show up in the intensity map or the spin surface. Since experiments are almost always performed along some high-symmetry lines and planes, we slide through several traditional high symmetry surfaces. Figure 1 displays the spin surfaces within four planes, where the false colors represent the amount of spin moment changes with the color bars depicting their true values. In Figs. 1(a)–1(d), five curves numbered from 1 to 5 represent their Fermi surfaces. Our results are very insightful. There are five spin surfaces that have an appreciable spin moment change; and other regions have a much smaller spin change. We start with the Γ-K-U-W-K plane (see Fig. 1(a)). Within this plane, the spin surface closely follows the Fermi surface (see Fermi surface 1 in the figure), but there is a distance between these two surfaces. This distance is a result of the laser photon energy 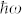. Depending on 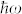, the spin surface can move away from or toward the Fermi surface. From the color, one can tell that the upper part of the spin surface is spin-moment-enhanced, while the lower part is spin-reduced. Within the same plane, another spin surface follows Fermi surface 2. This surface has the largest spin reduction in the entire BZ. The third spin surface follows Fermi surface 3, but the amount of the spin reduction is smaller than the first two.

Experimentally, Pickel *et al.*^6^ did detect the spin activities along the Δ direction. This is the predictive power of our theoretical method. However, Pickel *et al.*^6^ could not attribute those spin activities directly to the demagnetization, due to the reasons as discussed in the Introduction. Nevertheless, our theory does corroborate the spin change along the Δ direction. More importantly, our result reveals two much larger spin change pockets: one is slightly below the Δ line and close to the X point and the other is the largest spin change along the Λ direction and closer to the L point. Therefore, experimentally there is an opportunity to detect stronger spin signals. Future experiments can directly test our prediction.

Pickel *et al.*^6^ also theoretically showed additional spin hot spots along the Σ line, but this is not observed in our simulation. Instead, our data shows that there is no major spin change along that direction. This demonstrates that while a strong spin-orbit hybridization is important to the spin response, it is not a sufficient condition of a spin moment change. We also notice that in comparison with our band structure (see the bottom panel of Fig. 1) and prior experimental^12^ and theoretical results^19^,^20^, their band structure is unexpectedly upward-shifted by about 0.3 eV or the Fermi level is downshifted by the same amount. At present, we do not know the reason behind this discrepancy.

Figure 1(b) shows the spin dispersion in the L-W′-U-K plane. Due to SOC, W and W′ points become nonequivalent. The major spin changes appear in two different regions. The first one is around the L point, and the second one is around the W point. Different from Fig. 1(a), here the spin reduction and spin enhancement appear in the same region. However, the spin reduction surface is closer to Fermi surface 3, but the spin enhancement surface is further away from Fermi surface 3. On the other hand, the spin surface around the W points only shows the spin decrease. In general, this plane has more points with spin moment reduced than those with spin enhanced.

Figure 1(c) illustrates the spin change in a plane that is perpendicular to the Δ line. The spin surface (a square) differs from the Fermi surface 5 (a circle). The spin change is the smallest among these four planes. Different from Fig. 1(c), Fig. 1(d) shows a stronger spin reduction. This plane is perpendicular to the **k***_z_* axis. The main changes are closer to the W points.

In order to understand the above results, we first note that since the photon energy is fixed, there are only a limited number of the states that can be excited strongly. This can be seen from the band structure itself (bottom panel of Fig. 1). For instance, close to Γ point, transitions are difficult since there is no state available around 1.9 eV. Second, without SOC, the optical transition conserves the spin and no spin change should be observed. This also applies to the Coulomb and exchange interactions, 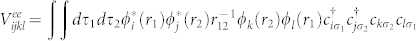, where *r*_12_ is the distance between two electrons. Electrons can stay in any orbitals *i* through *l*, but they can only carry two different spin indices *σ*_1_ and *σ*_2_. The fundamental reason is because the electrostatic interaction does not operate on the electron spins. Mathematically, *V_ee_* commutes with the total spin^32^. This has an important consequence on the Stoner excitation. Although the Stoner excitation is frequently cited as a way to the demagnetization, its demagnetization also relies on a spin symmetry breaking term, which is often SOC. However, the traditional theory hides this origin by invoking a thermal reservoir^9^. Third, with SOC, spin moment changes become possible. Our data shows that the most important contribution to the spin moment change comes from those transitions with a bigger difference between initial and final spin moments. The challenge is that these transitions are not optically favorable. In other words, transitions with a large spin change are often weakly dipole-allowed. On the other hand, those strongly dipole-allowed transitions have smaller spin changes. As a result, the true demagnetization as seen in these planes is a compromise between these two competing effects, which is characterized by the spin-dipole factor^10^,^34^.

Figure 2(a) shows the three-dimensional spin moment dispersion in the crystal momentum space. This is an isosurface with spin moment change of −0.3 *μ*_B_ which contributes demagnetization. This dispersion is constructed from 73763 irreducible **k** points, among which only 681 **k** points with the spin moment change are smaller than −0.15 *μ*_B_ (more negative). For our present laser parameters, the largest moment change is −0.72 *μ*_B_ at (46, 44, −44)/104 (in the unit of 2*π*/*a*, where *a* is the lattice constant of Co). If all the other **k** points had the similar spin reduction, this would mean 56% reduction in the spin moment. Unfortunately, a large portion of **k** points are silent. In addition, not all the **k** points have their spin moments reduced, and some **k** points see an increase in spin moment. For instance, the largest increase in spin moment is +0.44 *μ*_B_ at (76, 22, 0)/104. This decreases the amount of the spin reduction. The key message from this figure is that the spin change is not evenly distributed and is well localized behind the L-U-W and the hexagonal planes which are consistent with our above discussion. To help locate these spin isosurfaces, in Figs. 2(b) and 2(c) we show the corresponding Fermi surfaces. They closely resemble each other. Future experiments can use the Fermi surface as a guide to detect those spin isosurfaces. If these isosurfaces are measured as a function of time, one can construct a dynamic three dimensional crystal-momentum-resolved dispersion of the spin moment change, a movie in real time domain. Our Supplementary Material shows our theoretical movie. An experimental verification is much needed.

In conclusion, for the first time the ultrafast spin moment change is dispersed in the entire Brillouin zone for fcc Co. Our theory finally links those spin activities observed experimentally to the genuine spin moment change. Our results support the experimental observation of the strong spin activities along the Δ line^6^. More importantly, we reveal two even stronger but previously unobserved spin hot pockets. One pocket is slightly below the Δ line and the other is along the Λ line. This presents an excellent opportunity for future experiments. Further developments in experimental techniques will finally allow one to probe the dispersion of spin moment dispersion **k** point by **k** point, a necessary step to fully understand femtosecond magnetism.

## Author Contributions

M.S.S., J.Y.L., D.Z.Y. and G.P.Z. designed and performed the research; M.S.S., D.S.X. and G.P.Z. wrote the paper.

## Supplementary Material

Supplementary Information Supplementary video 1

Supplementary Information Supplementary video 2

Supplementary Information Supplementary video 3

Supplementary Information Supplementary video 4

Supplementary Information Supplementary material

## Figures and Tables

**Figure 1:**
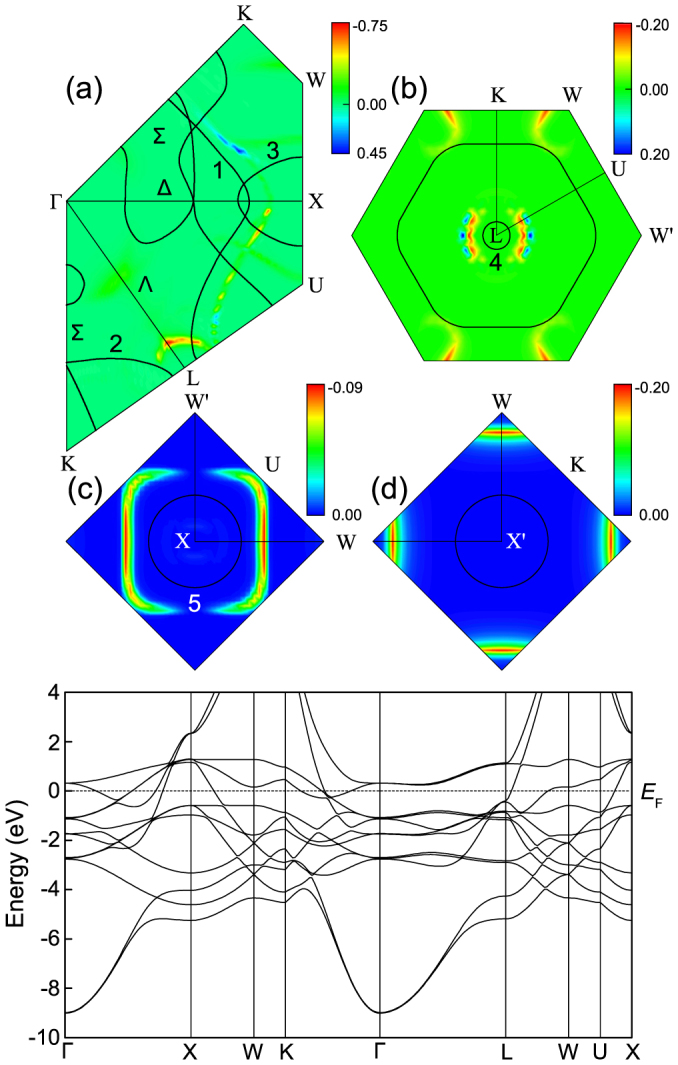
(a) Spin moment change 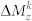 in the Γ-X-W-K and Γ-X-U-L-K planes. The intensity map denotes the spin moment change (in units of *μ*_B_). The curves denote the Fermi surfaces (same for all the other figures). The spin surfaces follows the Fermi surfaces. (b) Spin moment changes in the L-K-W-U-W′ plane are concentrated around Fermi surface 4. (c) Spin reduction in the X-W-W′ plane is the smallest among those four planes. (d) Spin moment change in the X′-W-W plan. The spin surface is away from the Fermi surface. Bottom panel: Band structure of fcc Co.

**Figure 2:**
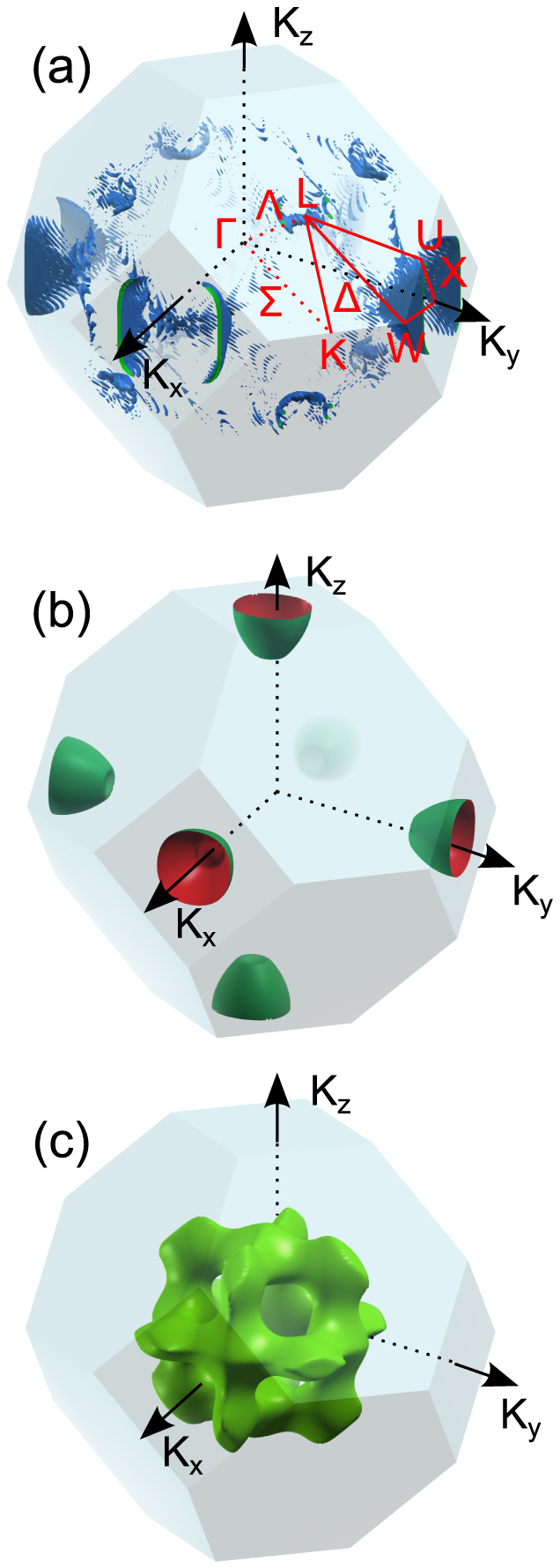
(a) Average spin moment change (averaged after 90 fs) is dispersed in the Brillouin zone. The isosurface value is taken at −0.3 *μ*_B_. The high-symmetry points and lines are labeled. (b) and (c) Two selected Fermi surfaces which are related to the isospin surfaces closely.

## References

[b1] Cortes R. *et al.* Momentum-resolved ultrafast electron dynamics in superconducting Bi_2_Sr_2_CaCu_2_O_8+*δ*_. Phys. Rev. Lett. 107, 097002 (2011). 21929262 10.1103/PhysRevLett.107.097002

[b2] Petersen J. C. *et al.* Clocking the melting transition of charge and lattice order in 1*T*-TaS_2_ with ultrafast extreme-ultraviolet angle-resolved photoemisssion spectroscopy. Phys. Rev. Lett. 107, 177402 (2011). 22107580 10.1103/PhysRevLett.107.177402

[b3] Rettig L. *et al.* Ultrafast momentum-dependent response of electrons in antiferromagnetic EuFe_2_As_2_. Phys. Rev. Lett. 108, 097002 (2012). 22463660 10.1103/PhysRevLett.108.097002

[b4] Aeschlimann M. *et al.* Ultrafast spin-dependent electron dynamics in fcc Co. Phys. Rev. Lett. 79, 5158–5161 (1997).

[b5] Goris A. *et al.* Role of spin-flip exchange scattering for hot-electron lifetimes in cobalt. Phys. Rev. Lett. 107, 026601 (2011). 21797630 10.1103/PhysRevLett.107.026601

[b6] Pickel M. *et al.* Spin-orbit hybridization points in the face-centered-cubic cobalt band structure. Phys. Rev. Lett. 101, 066402 (2008). 18764479 10.1103/PhysRevLett.101.066402

[b7] Weber A. *et al.* Ultrafast demagnetization dynamics of thin Fe/W(110) films: Comparison of time- and spin-resolved photoemission with time-resolved magneto-optic experiments. Phys. Rev. B 84, 132412 (2011).

[b8] Beaurepaire E., Merle J.-C., Daunois A. & Bigot J.-Y. Ultrafast spin dynamics in ferromagnetic nickel. Phys. Rev. Lett. 76, 4250–4253 (1996). 10061239 10.1103/PhysRevLett.76.4250

[b9] Zhang G. P. *et al.* Ultrafast spin dynamics in ferromagnetic nickel. Topics Appl. Phys. 83, 245–288 (2002).

[b10] Si M. S., Li J. Y., Xue D. S. & Zhang G. P. Manipulating femtosecond magnetism through pressure: First-principles calculations. Phys. Rev. B 88, 144425 (2013).

[b11] Ross C. A. Preface: Proceedings of the 56th annual conference on magnetism and magnetic materials, Scottsdale, Arizona, USA, October-November 2011. J. Appl. Phys. 111, 07A101 (2012).

[b12] Schneider C. M. *et al.* Curie temperature of ultrathin films of fcc-cobalt epitaxially grown on atomically flat Cu(100) surfaces. Phys. Rev. Lett. 64, 1059–1062 (1990). 10042152 10.1103/PhysRevLett.64.1059

[b13] Harp G. R. *et al.* Unusual stability of fcc Co(110)/Cu(110). Phys. Rev. B 48, 17538–17544 (1993). 10.1103/physrevb.48.17538 10008369

[b14] Guo G. Y. & Wang H. H. Gradient-corrected density functional calculation of elastic constant of Fe, Co and Ni in bcc, fcc and hcp structure. Chin. J. Phys. 38, 949–961 (2000).

[b15] Lefkidis G., Zhang G. P. & Hübner W. Angular momentum conservation for coherently manipulated spin polarization in photoexcited NiO: An *ab initio* calculation. Phys. Rev. Lett. 103, 217401 (2009). 20366066 10.1103/PhysRevLett.103.217401

[b16] Blaha P., Schwarz K., Madsen G. K. H., Kvasnicka D. & Luitz J. WIEN2k, An Augmented Plane Wave + Local Orbitals Program for Calculating Crystal Properties (Karlheinz Schwarz, Techn. Universität Wien, Austria, 2001).

[b17] Zhang G. P., Bai Y. & George T. F. Energy- and crystal momentum-resolved study of laser-induced femtosecond magnetism. Phys. Rev. B 80, 214415 (2009).

[b18] Perdew J. P., Burke K. & Ernzerhof M. Generalized gradient approximation made simple. Phys. Rev. Lett. 77, 3865–3868 (1996). 10062328 10.1103/PhysRevLett.77.3865

[b19] Moruzzi V. L., Janak J. F. & Williams A. R. Calculated electronic properites of metals. Pergamon, New York, (1978).

[b20] Himpsel F. J. & Eastman D. E. Experimental energy-band dispersions and magnetic exchange splitting for cobalt. Phys. Rev. B 21, 3207–3213 (1980).

[b21] Si M. S. & Zhang G. P. Resolving photon-shortage mystery in femtosecond magnetism. J. Phys.: Condens. Matter 22, 076005 (2010). 21386402 10.1088/0953-8984/22/7/076005

[b22] Sharma S., Dewhurst J. K. & Ambrosch-Draxl C. Linear and second-order optical response of III-V monolayer superlattices. Phys. Rev. B 67, 165332 (2003).

[b23] Ambrosch-Draxl C. & Sofo J. O. Linear optical properties of solids within the full-potential linearized augmented planewave method. Comp. Phys. Commun. 175, 1–14 (2006).

[b24] Steiauf D. & Fähnle M. Elliott-Yafet mechanism and the discussion of femtosecond magnetization dynamics. Phys. Rev. B 79, 140401(R) (2009).

[b25] Carva K., Battiato M. & Oppeneer P. M. Ab initio investigation of the Elliott-Yafet electron-phonon mechanism in laser-induced ultrafast demagnetization. Phys. Rev. Lett. 107, 207201 (2011). 22181762 10.1103/PhysRevLett.107.207201

[b26] Battiato M., Carva K. & Oppeneer P. M. Superdiffusive spin transport as a mechanism of ultrafast demagnetization. Phys. Rev. Lett. 105, 027203 (2010). 20867735 10.1103/PhysRevLett.105.027203

[b27] Eschenlohr A. *et al.* Ultrafast spin transport as key to femtosecond demagnetization. Nat. Mater. 12, 332–336 (2013). 23353629 10.1038/nmat3546

[b28] Mentink J. H. *et al.* Ultrafast spin dynamics in multisublattice magnets. Phys. Rev. Lett. 108, 057202 (2012). 22400955 10.1103/PhysRevLett.108.057202

[b29] Malinowski G. *et al.* Control of speed and efficiency of ultrafast demagnetization by direct transfer of spin angular momentum. Nat. Phys. 4, 855–858 (2008).

[b30] Boeglin C. *et al.* Distinguishing the ultrafast dynamics of spin and orbital moments in solids. Nature 465, 458–461 (2010). 20505724 10.1038/nature09070

[b31] Zhang G. P., Hübner W., Lefkidis G., Bai Y. & George T. F. Paradigm of the time-resolved magneto-optical Kerr effect for femtosecond magnetism. Nature Phys. 5, 499–502 (2009).

[b32] Zhang G. P. & Hübner W. Laser-induced ultrafast demagnetization in ferromagnetic metals. Phys. Rev. Lett. 85, 3025–3028 (2000). 11005994 10.1103/PhysRevLett.85.3025

[b33] Zhang G. P., Lefkidis G., Hübner W. & Bai Y. Ultrafast demagnetization in ferromagnets and magnetic switching in nanoclusters when the number of photons is kept fixed. J. Appl. Phys. 109, 07D303 (2011).

[b34] Si M. S. & Zhang G. P. Hot spin spots in the laser-induced demagnetization. AIP Advcances 2, 012158 (2012).

