# Supplementary material for: Crystal-momentum dispersion of ultrafast spin change in fcc Co
Source: Sci Rep. 2014 May 23;4:5010. doi: 10.1038/srep05010 (PMC4031477; doi:10.1038/srep05010)
Supplement: Supplementary Information — Supplementary material [file srep05010-s5.pdf]

Supplementary Material of "Crystal-momentum dispersion of ultrafast spin change in fcc Co"

M. S. Si

Key Laboratory for Magnetism and Magnetic Materials of the Ministry of Education, Lanzhou University, Lanzhou 730000, China, &

Department of Physics, Indiana State University, Terre Haute, Indiana 47809, USA

J. Y. Li, D. Z. Yang, and D. S. Xue

Key Laboratory for Magnetism and Magnetic Materials of the Ministry of Education, Lanzhou University, Lanzhou 730000, China

G. P. Zhang

Department of Physics, Indiana State University, Terre Haute, Indiana 47809, USA

(Dated: February 17, 2014)

In the main paper, we focus attention on the time-averaged spin moment change after 90 fs. Since the spin moment change is not only momentum-dependent, but also time-dependent, in this Supplementary Material we will complete this part. We show that the time evolution of spin moment change can reveal additional insights into femtomagnetism. The time-resolved spin moment change reads

$$\Delta M_z^{\mathbf{k}}(t) = M_z^{\mathbf{k}}(t) - M_z^{\mathbf{k}}(-\infty), \tag{1}$$

where $M_z^{\mathbf{k}}(t)$ and $M_z^{\mathbf{k}}(-\infty)$ are the spin moments at time t and at a negative infinite time without any laser influence, respectively. We construct four movies to show the time-resolved spin moment change in the crystal momentum space. Movies from S1.avi to S4.avi correspond to Figs. 1(a)-1(d) in the main paper.

S1.avi shows the time evolution of spin moment change in the Γ-X-W-K and Γ-X-U-L-K planes. Before -0.27 fs, no obvious hot spin spots [1] is detectable. After that, three hot spin spots occur simultaneously and form active centers of spin surfaces 1-3: (1) On spin surface 1, a hot spin spot appears slightly above the Δ line close to the X point; (2) On spin surface 2, it is located along the Λ line close to the L point; (3) On spin surface 3, it occurs along the W-L line close to the X point. As time goes on, these earliest hot spin spots are enhanced. Very soon, these hot spin spots grow outward along their respective Fermi surfaces. Under the continuous photon excitation of laser field, these hot spin spots eventually form the spin surfaces, similar to their corresponding Fermi surfaces. But, there is still a distance between them since a finite photon energy is used in the simulation [2]. As stated in the main paper, only three Fermi surfaces 1 through 3 are associated with the spin surfaces 1 through 3.

In contrast to spin surfaces 2 and 3, spin surface 1 looks more like the Fermi surface 1. It forms a stretched crescent. Spin surface 2 appears to be a more distorted crescent localized near the L point, and spin surface 3 has a crescent structure. More importantly, all of them closely follow their respective Fermi surfaces. These Fermi surfaces serve as a guide experimentally to probe the spin surfaces.

It is surprising that two hot spin enhancement spots also appear in spin surface 1 at ∼4.84 fs. They are separately distributed in the middle region of the Γ-X-W-K plane and in the lower region of Γ-L-U-X plane close to the L point, respectively. This implies that the spin surface consists of both hot spin reduction spots and hot spin enhancement spots, which

stems from the different spin expectation value change induced by the electron transitions.

At time around 26.33 fs, spin surfaces 1-3 reach their maximums, which coincides with the maximum demagnetization occurring at this moment in the magnetization curve. It is interesting that the spin surface itself is not uniform and consists of the discrete domains. For instance, near the Λ line spin surface 2 is split into three very localized hot spin spots. The level of the discreteness is strongest in spin surface 3 as more hot spin spots appear near the U point. This continuous-to-discrete transition in spin surfaces depends on time, which has been never reported in the literature. The physics origin can be well understood by the spin-dipole factor [1], where the ultimate spin change is determined by a product of the dipole transition moment and the intrinsic spin moment change.

After the maximum demagnetization, the spin surfaces oscillate around its equilibrium value. Our finding is very insightful. Two main factors contribute such an oscillation: One originates from the oscillated intensity of spin moment change; another comes from the variation between the continuous-discrete spin moment change, which is ascribed to the electron transitions mediated by the laser field. Future experiment can test the above prediction directly.

Next, we discuss the time-resolved spin moment change in the L-K-W-U-W$'$ plane, as illustrated in S2.avi. In contrast to the Γ-X-W-K and Γ-X-U-L-K planes, the first emergence of spin activities in this plane is the hot spin enhancement spots at around -4.36 fs. These hot spin spots form two facing crescents which are centered at the L point. They are symmetrically distributed on both sides of the L-K line. Interestingly, it surrounds the Fermi surface 4. For convenience, we label it as spin surface 4 because of its similar shape to the Fermi surface 4.

When the time is close to about -0.27 fs, two regions of hot spin reduction spots simultaneously start in this plane: One forms an L shaped pocket near each W point; another appears more complex as it is involved in the formation of spin surface 4. As can be seen, two parts compose the latter hot spin reduction spots in spin surface 4. One part forms four solid circles, locating near the ends of two facing crescents, while another part is more dispersive, which is close to the inner margin of spin surface 4. Though the inner part is weaker than the four solid circles, it has a big effect on the two facing crescents' spin pocket, changing the original rounded structure to an edged one.

As time further progresses, spin surface 4 looks more like a complete circle. In contrast

to Fermi surface 4, spin surface 4 appears more dispersive and has a thicker circle's width. It possesses two circles with hot spin reduction spots separated by the spin enhancement pocket. Though the configuration of spin surface 4 is nearly identical to the Fermi surface 4, there is still a distance between them. As stated in Ref. [2], this distance can be tuned through varying the photon energies used in the simulation.

If we take a closer look at the spin moment change in the demagnetization process before the maximum demagnetization time around 26.3 fs, we find that the spin pockets near the W points increase monotonously with time. But spin surface 4 has a more complicated time-dependence: It first increases in the intensity (more negative for the hot spin reduction spots and more positive for the hot spin enhancement spots), and then decreases, followed by an increase again. The net magnetization change from this plane is determined by a competition between the spin surface 4 and the spin pockets near the W points. This is the case for other momentum planes as well.

After the maximum at ∼26.3 fs, the spin moment change slightly recovers its value all the way up to 52 fs (see the magnetizaton curve in the left panel of S2.wmv). This results in a reduction in the spin pockets near the W points, but a continuous enhancement in spin surface 4. At about 52 fs, the spin surface 4 reaches its maximum. More importantly, some strong localized spin enhancement spots start to appear, which will affect the following magnetization dynamics.

When the magnetization undergoes the second reduction after ∼52 fs, a very impressive feature appears. Spin pockets near the W points still increase in their amplitudes, while the spin surface 4 is discretized with a nearly unchanged amplitude. The outer circle of spin surface 4 is developed into eight regular hexagons with the strongest hot spin reduction spot at each hexagonal center. In contrast to the outer circle, the inner circle of spin surface 4 is nearly featureless. However, as time is close to around 76 fs, this inner spin pocket evolves into six clover shaped spin pockets.

When the spin moment change oscillates around its equilibrium value at about 110 fs, the outer eight hexagons further evolve into four rectangular structures which are located along the L-U lines. Obviously, two distinct time-dependent features are involved in the spin surface 4. We emphasize this intrinsic character of spin moment change is momentum-dependent. It should be the focus of future research.

Due to the broken symmetry related to the spin-orbit coupling in fcc Co, the quadran-

gle planes perpendicular to $\mathbf{k}_z$ and $\mathbf{k}_x$ or $\mathbf{k}_y$ directions are not equivalent. This causes a direct symmetry breaking in the spin moment change. In the following, we study the time evolution of spin moment change in these two quadrangle planes to address this point. The corresponding movies are referred to the S3.avi and S4.avi, respectively.

In the X-W-W$'$ plane, the spin moment change forms a quadrangle around 1.77 fs. It has a very similar shape to that of Fermi surface 5. We denote it as spin surface 5. Because of the presence of spin-orbit coupling in this plane, W and W$'$ points are inequivalent again. As a result, the hot spin spots appear along the X-W line, but not along the X-W$'$ line. During the entire demagnetization process before 26 fs, spin surface 5 increases monotonously in its intensity. The strongest hot spin spot in this spin surface is along the X-W line. As the magnetization starts to oscillate, the discrete structure is also observed at the four corners of spin surface 5, which are near the U and U$'$ points.

Finally, we show the time-resolved spin moment change in the X$'$-W-W plane. In this plane, hot spin spots are very localized mainly near the W points. Each of them is a straight line segment. These spin pockets are decoupled from the Fermi surface as there is a very large distance between them. Particularly, these spin pockets do not closely follow the Fermi surface in time. No spin surface is in this plane. Therefore, the above proposed method to detect spin surface based on the corresponding Fermi surface becomes a big challenge. In comparison with the other planes, these spin pockets are less discrete.

In summary, we show that the time-resolved spin moment change sensitively depends on both the crystal momentum and time. In the case of Γ-X-W-K and Γ-X-U-L-K planes, the hot spin spots form spin surfaces 1-3, which are restricted to a small part of each spin surface. In the L-K-W-U-W$'$ plane, spin surface 4 starts with a few spin enhancement spots, but when the magnetization starts to oscillate, it is strongly discretized. In the same plane, one spin pocket with an L shape appears near each W point. In the X-W-W$'$ plane, spin surface 5 is a quadrangle, which is asymmetric with respect to the W and W$'$ points due to the spin-orbit coupling. No spin surface can be detectable in the X$'$-W-W plane. But the very localized hot spin spots form a line segment near the W points. Our main finding is summarized in Table I. This can be used as a good guide to future experimental

investigations.

[1] Si, M. S. & Zhang, G. P. Hot spin spots in the laser-induced demagnetization. *AIP Advances* **2**, 012158 (2012).

[2] Si, M. S., Li, J. Y., Xue, D. S. & Zhang, G. P. Manipulating femtosecond magnetism through pressure: First-principles calculations. *Phys. Rev. B* **88**, 144425 (2013).

PACS numbers: 75.40.Gb, 78.20.Ls, 75.70.-i, 78.47.J-

TABLE I: Nature of time-resolved spin moment change in fcc Co.

Plane	Γ-X-W-K<br>Γ-X-U-L-K	L-K-W-U-W′	X-W-W′	X′-W-W
Main feature	sporadic islands	facing crescents<br>L-shapes	quadrangle	line segment
